# Risk of venous thromboembolism in elderly patients with vertebral compression fracture: A population-based case–control study

**DOI:** 10.1097/MD.0000000000020072

**Published:** 2020-05-01

**Authors:** Ching-Hui Huang, Wei-Hsun Wang, Chew-Teng Kor, Ching-Hua Hsiao, Chia-Chu Chang

**Affiliations:** a Division of Cardiology, Department of Internal Medicine, Changhua Christian Hospital, Changhua; b School of Medicine, College of Medicine, Kaohsiung Medical University, Kaohsiung; c Department of Beauty Science and Graduate Institute of Beauty Science Technology, Chienkuo Technology University; d Department of Orthopedic, Changhua Christian Hospital, Changhua; e Medical Research Center, Department of Internal Medicine, Changhua Christian Hospital, Changhua; f Section of Joint Reconstruction, Department of Orthopedic, Kuang Tien General Hospital; g Department of Internal Medicine, Kuang Tien General Hospital; h Department of Nutrition, Hungkuang University, Taichung, Taiwan.

**Keywords:** elderly, percutaneous vertebroplasty, venous thromboembolism, vertebral compression fractures

## Abstract

Vertebral compression fractures (VCFs) are common in elderly and are treated with immobilization. Moreover, immobilization and old age may increase venous thromboembolism (VTE) risk. However, the incidence of VCFs-related VTE is unknown in elderly. The purposes of this study were to determine the incidence of VTE among VCF patients, to explore whether percutaneous vertebroplasty (PV) intervention may reduce VTE risk in VCFs patients.

We conducted a population-based case–control study by using the National Health Insurance Research Database. We identified 1407 patients aged ≥65 with VCF who received PV and 1407 VCFs patients who did not receive PV after developing a 1:1 propensity score-matched study cohort and were followed up for 5 years. Using PV intervention as the exposure factor, a cause-specific Cox's proportional hazards model was used to examine the association between PV and VTE.

After propensity score matching, the mean age of the study participants was 78 years and ∼23% of the analyzed participants were men, incidence of VTE in the PV and control cohorts was 5.77 and 4.19 per 1000 person-years, respectively. Both groups were nonsignificant difference after examination with different adjustment models. Patients with VCF and a history of heart failure, coronary artery disease, receiving antihypertension medication were at a significantly increased VTE risk.

Elderly patients with VCF who received PV had a neutral impact on risk of VTE. VCF patients with heart failure, coronary artery disease, and receiving antihypertension medication were prone to developing VTE should be monitored cautiously.

## Abstract 2

Supplemental Digital Content is available in the text

## 1 Introduction

Incidence of osteoporotic vertebral compression fracture (VCF) increases with age.^[1]^ VCFs are often highly painful and are treated with immobilization.^[1]^ Moreover, immobilization and old age may increase venous thromboembolism (VTE) risk in hospitalized patients.^[2,3]^ VTE presents clinically as deep vein thrombosis (DVT), pulmonary embolism (PE), or both. VTE contributes to considerable morbidity and mortality and causes a huge socioeconomic burden.^[4]^ Percutaneous vertebroplasty (PV) is a minimally invasive, therapeutic, and interventional radiological procedure involving injection of bone cement (methyl methacrylate) into a fractured vertebral body; this procedure is indicated for painful osteoporotic VCFs refractory to medical therapy.^[5]^ PV can increase patient mobility, provide pain relief, and prevent further vertebral collapse with potential for improving functional outcomes.^[6]^ However, the incidence of VCFs-related VTE is unknown and data about which comorbidities prone to developing VTE in elderly VCFs patients are limited.^[7]^ We hypothesized that elderly VCF patients who received PV intervention can provide pain relief and increase patient mobility, which decrease the risk of VTE. Therefore, we conducted a cohort study with 5 yeas follow-up duration to evaluate PV efficacy and safety on VTE risk and explore VTE prevalence among elderly patients with VCF. This population-based cohort study investigated the epidemiological and risk factors for VTE in patients with VCF who had and had not received PV in Taiwan based on data from the National Health Insurance Research Database (NHIRD)

## 2 Methods

### 2.1 Data source and study patients

Data were retrieved from the NHIRD, which contains all claims data from the National Health Insurance (NHI) program from 2000 to 2013. One million people were randomly sampled from those enrolled in the Longitudinal Health Insurance Database (LHID) 2005—a subset of the NHIRD. We identified patients with VCF diagnoses in the LHID 2005 population from 2000 to 2013. Diseases were identified based on International Classification of Diseases, Ninth Revision, Clinical Modification (ICD-9-CM) codes. This study was approved after a full ethical review by the Institutional Review Board (IRB) of the Changhua Christian Hospital (approval number 180205). The IRB waived the need for consent. Data were accessed anonymously.

**Figure 1:**
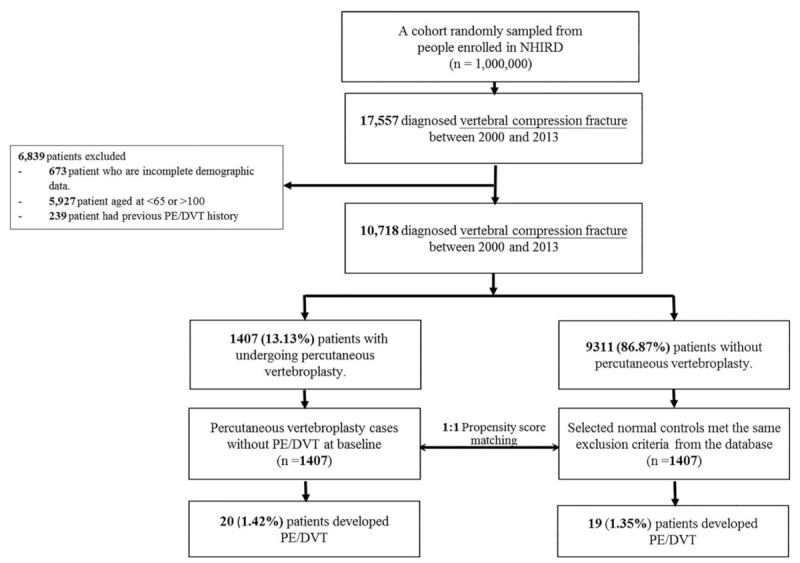
Study flowchart.

A flowchart of the subject selection process is shown in Figure 1. Patients diagnosed with VCF (ICD-9-CM codes 805.2, 805.3, 805.4, 805.5, 806, and 733.13) and with at least three records in the claims data from January 1, 2000, to December 31, 2013, were included in this study. The index date was the date of VCF diagnosis. Patients aged <65 years or >100 years or with incomplete demographic information were excluded. Patients diagnosed with PE (ICD-9-CM code 415.1) or DVT (ICD-9-CM code 453.8) before the index date were also excluded. VTE (PE or DVT) diagnosis was required to meet one of the following criteria:

1. one or more inpatient admissions with VTE diagnosis and
2. at least three records of VTE diagnosis after the index date.

In total, 10,718 patients diagnosed with VCF were included. Among these patients, 9311 (86.87%) did not receive PV and 1407 (13.13%) received PV. To remove covariate imbalance, one propensity score-matched study cohort was established. Based on the propensity score, 1:1 propensity score matching was performed in our study cohort. Propensity scores were calculated using multivariate logistic regression to predict the probability of VTE. Each identified patient with VCF who received PV was propensity score matched to a patient with VCF who did not receive PV. In total, 1407 patients with VCF who received PV and 1407 propensity score-matched patients with VCF who did not receive PV were included in the study cohort. The details of the propensity score model are described in Table S1.

### 2.2 Outcome measures and relevant variables

#### 2.2.1 Exposure to vertebral augmentation procedures

Vertebral augmentation procedures involving vertebroplasty were identified using NHI procedure codes (33126B for PV [first vertebra] and 33127B for PV [any vertebra after the first]) or ICD-9-CM billable procedure codes (81.66 for percutaneous vertebral augmentation and 81.65 for PV). Patients who received the aforementioned procedures were defined as the PV group. Patients with VCF who did not receive the aforementioned procedures were defined as control group.

#### 2.2.2 Other relevant variables

Major comorbid diseases diagnosed in at least three records in the claims data within 1 year before the index date were defined as baseline comorbidities. Comorbidities included hypertension, diabetes mellitus, hyperlipidemia, chronic kidney disease (CKD), chronic obstructive pulmonary disease (COPD), stroke, cardiac dysrhythmia, peripheral artery occlusive disease (PAOD), all cancers, coronary artery disease (CAD), and congestive heart failure (CHF). Additionally, long-term medications thought to be associated with VTE, including diabetic drugs, statins, analgesics, antihypertensive drugs, and anticoagulation drugs, were assessed using the NHIRD.

#### 2.2.3 Statistical methods

The demographic and clinical characteristics of patients with VCF who received vertebral augmentation procedures and those who did not were summarized using numbers and percentages for the categorical variables and means ± standard deviations for continuous variables. Chi-squared and *t* tests were conducted to compare the distribution of discrete and continuous variables, respectively. Cox proportional hazards models were used to estimate the relative VTE risk in patients with VCF who received PV and those who did not. Confounders, including age, monthly income, major comorbidities, and long-term medication use, were adjusted in multivariate Cox analysis to estimate adjusted hazard ratios (aHRs). Because comorbid diseases may not only be present at the baseline but also develop during the follow-up period, such diseases were modeled using nonreversible time-dependent binary covariates for event analyses. A two-tailed *P* value of <.05 was considered statistically significant. All statistical analyses were performed using IBM SPSS Statistics for Windows, version 22.0 (IBM Corp., Armonk, NY).

## 3 Results

### 3.1 Characteristics of the study population

The characteristics of the study population are listed in Table 1 for comparison. Regarding the patients before propensity score matching, consistent differences existed among the baseline characteristics of patients with VCF who received PV and those who did not. Those who did were more likely to be older and have a lower monthly income than those who did not. Furthermore, the patients who received PV were more likely to have hypertension, hyperlipidemia, diabetes mellitus, CKD, stroke, COPD, CAD, heart failure, cardiac arrhythmia, and a higher Charlson comorbidity Index score than were those who did not receive PV. Moreover, medications including diabetic drugs, statins, analgesics, and antihypertensive drugs were more frequently prescribed to the patients who received PV than to those who did not. The percentages of patients using anticoagulants were comparable in both groups. In the propensity score-matched model, age, sex, and monthly income were similar in both groups. Furthermore, the prevalence of comorbidities, including hypertension, hyperlipidemia, diabetes mellitus, obesity, heart failure, and stroke, was similar in both groups. Both groups used diabetic drugs, statins, analgesics, antihypertensive drugs, and anticoagulation medications in a similar manner.

**Table 1:**
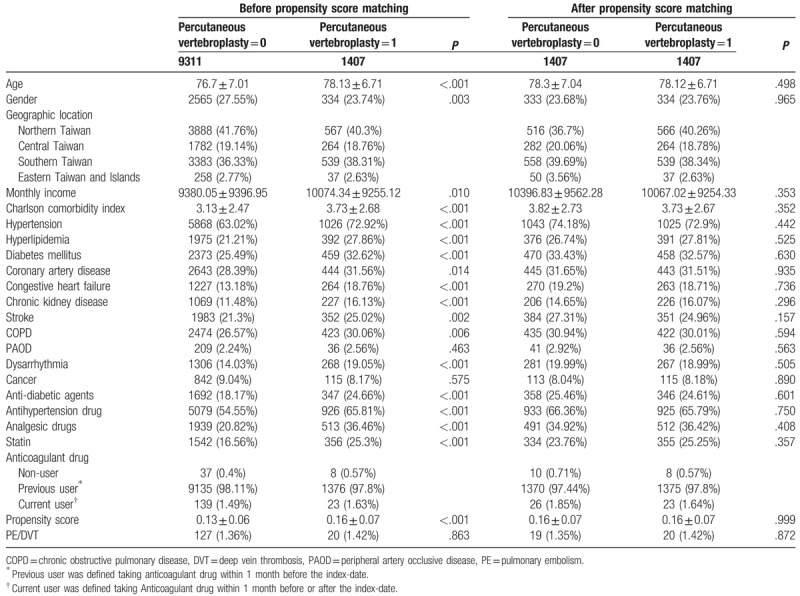
Demographic.

### 3.2 Association between VTE and patients with VCF who did and did not receive PV

**Figure 2:**
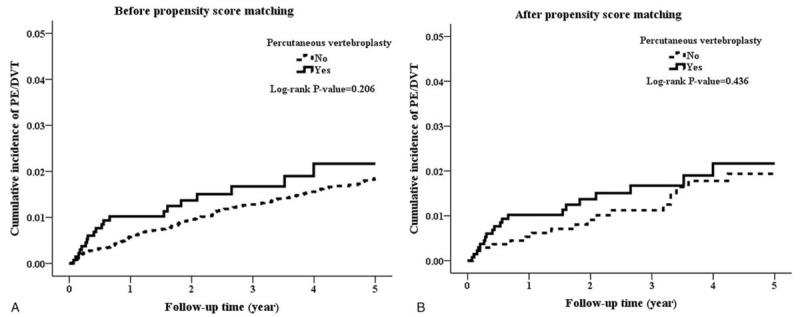
(A) Kaplan–Meier estimation for PE/DVT risk among patients who received (solid line) and did not receive (dashed line) PV before propensity score matching. (B) Kaplan–Meier estimation for PE/DVT risk among patients who received (solid line) and did not receive (dashed line) PV after propensity score matching.

Table 2 shows the results of the Cox proportional hazards model analysis regarding the role of VCF in predicting VTE. Before propensity score matching, incidence of VTE was higher nonsignificantly in the PV cohort than in the control cohort (5.77 vs 3.96 per 1000 person-years, crude hazard ratio [cHR]: 1.36, 95% confidence interval [CI]: 0.84–2.18, *P* = .207). Furthermore, in the propensity score-matched data, the Cox proportional hazards model analysis demonstrated that susceptibility to VTE was similar in both groups (cHR: 1.28, 95% CI: 0.68–2.41, *P* = .438; Table 2). Incidence of VTE in the PV and control cohorts was 5.77 and 4.19 per 1000 person-years, respectively. After propensity score matching and multivariate adjustment, VTE risk was nonsignificantly higher in the PV cohort (aHR: 1.28, 95% CI: 0.68–2.41, *P* = .443) than in the control cohort. The results were consistent among various adjustment models (Table 2). Analysis of time-to-event outcomes using the Kaplan–Meier method for VTE risk among patients who did (solid line) and did not (dashed line) receive PV before and after propensity score matching is illustrated in Figure 2A and B. No significant difference in VTE rate during the 5-year follow-up period after propensity score matching was observed between the two groups in this analysis (*P* = .436, log rank test).

**Table 2:**
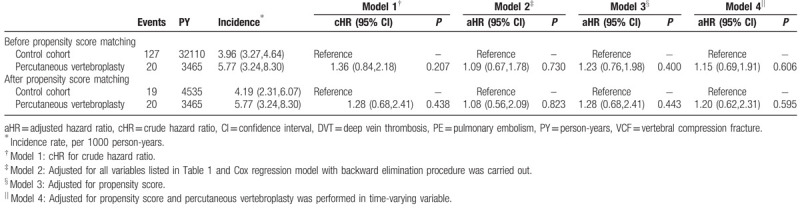
Incidence and risk of PE/DVT in VCF patients with percutaneous vertebroplasty and without percutaneous vertebroplasty intervention.

### 3.3 Comorbid disease status and risk of VTE in patients with VCF who did and did not receive PV

The results of subgroup analyses are shown in Table S2 Subgroup analyses were performed for sex, age <75 years, age ≥75 years, comorbidities <3, comorbidities ≥3, presence or absence of CHF, and anticoagulant drug used within 1 month after the index date in both cohorts. We observed that both cohorts had a similar VTE risk in all subgroups, regardless of propensity score matching.

### 3.4 Significant risk factors for VTE

To clarify the association between VCF and VTE in both cohorts, all subgroups were analyzed, as shown in Table 3. The multivariate Cox model indicated that patients with VCF who had CAD, CHF, and who received antihypertension drugs were more likely to develop VTE.

**Table 3:**
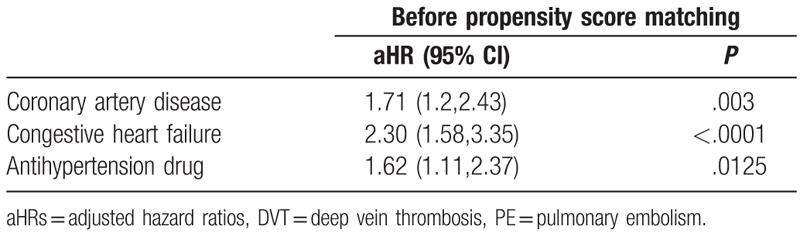
The significant risk factors of PE/DVT.

## 4 Discussion

The main finding of our study was that the incidence rates of VTE among patients with VCF were ∼4.19 per 1000 person-years in the control cohort and 5.77 per 1000 person-years in the PV cohort (*P* = .438) after propensity score matching. These results were confirmed using adjusted models. Our results were similar to those of one large retrospective cohort study conducted in the United Kingdom that used the General Practice Research Database^[8]^; that study reported annual incidence of VTE of 5.6 per 1000 person-years in untreated osteoporotic women with a mean age of 70 years. In our study, the mean age of the study sample was 78 years and ∼23% of the analyzed patients were men. VCF is the most common type of fragility fracture and is mainly due to osteoporosis; it is believed that VTE occurrence is lower among Asians than Caucasians.^[9]^ Although Asians are subject to the same major acquired risk factors for VTE as are Caucasians, studies conducted in Asia have consistently reported lower rates of VTE in Asians than Caucasians.^[10,11]^ By contrast, our study demonstrated no ethnic differences in VTE incidence among patients with VCF, regardless of PV treatment; this highlights the importance of VTE work-up in frail patients.

Patients with VCFs usually present with acute axial back pain, and pain following a VCF may be highly disabling and immobilizing, thereby increasing VTE risk among elderly patients with VCF. We hypothesized that PV might mitigate pain and allow patients with VCF greater mobility, thereby decreasing VTE risk. However, no significant difference in VTE incidence was observed between the PV and control groups. Previous studies have reported that PV must be injected at high pressure, and thus the risk of cement leakage is relatively great.^[12]^ VTE is reported as a procedural complication because bone cement leakage into the vertebral venous plexus may lead to late clinical manifestations of PE.^[13,14]^ According to the results of our study and a 5-year cohort study, PV intervention is not associated with higher VTE incidence among patients with VCF in various subgroups.

One study demonstrated that patients with heart failure had an increased rate of VCF,^[15]^ and given the increasing life expectancy of patients with heart failure, interventions to reduce fracture burden could improve overall quality of life and life expectancy in frail older populations. We investigated whether PV intervention could decrease VTE incidence among patients with VCF who had heart failure. Our study showed no significant difference of VTE incidence between the two groups. However, our data should be interpreted with caution because the heart failure sample size was relatively small. Moreover, heart failure was a significant risk factor for VTE in patients with VCF. Patients with heart failure showed an increased rate of VCF,^[15]^ and among patients with VCF, heart failure increased VTE risk 2.30 fold according to our study findings. Our findings could serve as a reminder for cardiologists to pay more attention to patients with heart failure who have VCF; patients with VCF are recommended to undergo screening for osteoporosis, take appropriate medicines to lower the risk of more serious fractures such as hip fractures, and undergo fall assessment. Adequate pain control is crucial for early mobilization in patients with heart failure who have VCF to reduce VTE risk. Furthermore, VCF with increased kyphosis can affect pulmonary function and could exacerbate heart failure symptoms.^[16]^

Policy implication of this study was to inform health providers and patients about the importance of prevention vertebral fractures through management of risk factors and the treatment of osteoporosis. Therefore, it is important to encourage weight-bearing and muscle-strengthening exercise for building and maintain bone density in elderly. Smoking cessation and avoidance of excessive alcohol consumption are recommended for bone health. Patients who are prone to osteoporosis should receive a *comprehensive falls*-*risk assessment*.

Our study had some limitations. First, body weight, biochemical data, and smoking history, all of which are crucial factors for osteoporosis, were not included. Second, bone mineral density score and image data of patients were not included in the study; therefore, the severity of osteoporosis and VCF could not be identified. This may have implications for study results. Further clinical studies are required to confirm our observations.

We concluded that incidence of VTE among patients with VCF was ∼4.19 per 1000 person-years in the control cohort and 5.77 per 1000 person-years in the PV cohort after propensity score matching. Patients with VCF who received PV had a neutral impact on risk of VTE. However, VCF patients with heart failure, coronary artery disease, and receiving antihypertension medication were prone to developing VTE should be monitored cautiously.

## Acknowledgments

This manuscript was edited by Wallace Academic Editing.

## Author contributions

**Acquisition of subjects and/or data:** Dr Ching-Hua, Hsiao, Dr Chew-Teng Kor, Dr Wei-Hsun Wang, and Dr Ching-Hui Huang

**Analysis and interpretation of data:** Dr Ching-Hui Huang, Dr Chew-Teng Kor, and Dr Wei-Hsun Wang

**Preparation of manuscript:** Dr Chia-Chu Chang, Dr Ching-Hui Huang, Dr Wei-Hsun Wang, and Dr Ching-Hua, Hsiao

**Study concept and design:** Dr Chia-Chu Chang and Dr Ching-Hua, Hsiao

## Supplementary Material

Supplemental Digital Content

## Supplementary Material

Supplemental Digital Content
