# Supplementary material for: Risk of venous thromboembolism in elderly patients with vertebral compression fracture: A population-based case–control study
Source: Medicine (Baltimore). 2020 May 1;99(18):e20072. doi: 10.1097/MD.0000000000020072 (PMC7440209; doi:10.1097/MD.0000000000020072)
Supplement: Supplemental Digital Content [file medi-99-e20072-s001.docx]

Table S1: The propensity-score model results of probability of venous thromboembolism

| Variables | Estimate | Odds ratio | 95% CI | | *P* value |
| --- | --- | --- | --- | --- | --- |
|  |  |  | Lower | Upper |  |
| Age | 0.0312 | 1.032 | 1.027 | 1.037 | <.0001 |
| Gender | -0.3027 | 0.739 | 0.652 | 0.837 | <.0001 |
| Index-year | 0.2597 | 1.296 | 1.272 | 1.321 | <.0001 |
| Geographic location |  |  |  |  |  |
| Northern Taiwan | 1 | -- | -- | -- | -- |
| Central Taiwan | 0.011 | 1.011 | 0.870 | 1.175 | 0.887 |
| Southern Taiwan | 0.144 | 1.155 | 1.021 | 1.306 | 0.022 |
| Eastern Taiwan and Islands | 0.083 | 1.087 | 0.765 | 1.544 | 0.642 |
| Comorbidity diseases at baseline |  |  |  |  |  |
| Hypertension | 0.1652 | 1.18 | 1.04 | 1.338 | 0.0101 |
| Congestive Heart Failure | 0.1875 | 1.206 | 1.027 | 1.417 | 0.0224 |
| COPD | 0.1613 | 1.175 | 1.033 | 1.337 | 0.0142 |
| Dysrhythmia | 0.153 | 1.165 | 0.997 | 1.363 | 0.0552 |
| Medication |  |  |  |  |  |
| Anti-diabetic agents | 0.1827 | 1.201 | 1.049 | 1.374 | 0.0081 |
| Analgesic drugs | 0.4709 | 1.601 | 1.419 | 1.807 | <.0001 |

Abbreviations: PAOD, peripheral artery occlusive disease; CI, confidence interval; COPD, chronic obstructive pulmonary disease.
