# Supplementary material for: Risk of venous thromboembolism in elderly patients with vertebral compression fracture: A population-based case–control study
Source: Medicine (Baltimore). 2020 May 1;99(18):e20072. doi: 10.1097/MD.0000000000020072 (PMC7440209; doi:10.1097/MD.0000000000020072)
Supplement: Supplemental Digital Content [file medi-99-e20072-s002.docx]

|  |
| --- |

Table S2. Subgroup analyses of the risk of venous thromboembolism in patients with percutaneous vertebroplasty and matched participants.

| Subgroup | Subjects without percutaneous vertebroplasty | | Subjects with percutaneous vertebroplasty | | Compared to control group | | | | |
| --- | --- | --- | --- | --- | --- | --- | --- | --- | --- |
|  | n | Event | n | Event | aHR (95% CI)§ | P-value | aHR (95% CI)^‡^ | P-value | P_interaction_ |
| **Before propensity score matching** | | | |  |  |  |  |  |  |
| Gender |  |  |  |  |  |  |  |  | 0.3062 |
| Female | 6746 | 118 | 1073 | 18 | 1.37(0.81,2.31) | 0.2382 | 1.39(0.82,2.37) | 0.2218 |  |
| Male | 2565 | 50 | 334 | 5 | 0.73(0.24,2.23) | 0.5785 | 0.78(0.25,2.40) | 0.6632 |  |
| Age, years |  |  |  |  |  |  |  |  | 0.8709 |
| <75 | 3844 | 62 | 433 | 7 | 1.29(0.50,3.31) | 0.5949 | 1.16(0.45,2.99) | 0.7538 |  |
| ≥75 | 5467 | 106 | 974 | 16 | 1.19(0.68,2.07) | 0.5421 | 1.21(0.69,2.11) | 0.5098 |  |
| Comorbidity |  |  |  |  |  |  |  |  | 0.5329 |
| <3 | 5460 | 77 | 671 | 5 | 0.91(0.36,2.29) | 0.8405 | 0.95(0.38,2.39) | 0.9077 |  |
| ≥3 | 3851 | 91 | 736 | 18 | 1.36(0.77,2.38) | 0.2898 | 1.33(0.75,2.35) | 0.3249 |  |
| Anticoagulant drug used within 1 month after the index-date | | | | |  |  |  |  | 0.5487 |
| Yes | 139 | 4 | 23 | 1 | 1.74(0.05,63.76) | 0.7623 | 1.13(0.04,31.98) | 0.3569 |  |
| No | 9172 | 164 | 1384 | 22 | 1.20(0.74,1.93) | 0.4634 | 1.25(0.78,2.03) | 0.9413 |  |
| Congestive Heart Failure | | |  |  |  |  |  |  | 0.9396 |
| Yes | 1227 | 43 | 264 | 9 | 1.04(0.48,2.26) | 0.918 | 1.27(0.58,2.77) | 0.5545 |  |
| No | 8084 | 125 | 1143 | 14 | 1.17(0.64,2.14) | 0.6204 | 1.16(0.63,2.15) | 0.6274 |  |
| **After propensity score matching** | | | |  |  |  |  |  |  |
| Gender |  |  |  |  |  |  |  |  | 0.3458 |
| Female | 1073 | 15 | 1072 | 18 | 1.58(0.77,3.21) | 0.2102 | 1.52(0.75,3.09) | 0.2507 |  |
| Male | 333 | 6 | 334 | 5 | 0.66(0.16,2.77) | 0.5722 | 0.70(0.17,2.91) | 0.6236 |  |
| Age, years |  |  |  |  |  |  |  |  | 0.7309 |
| <75 | 451 | 6 | 433 | 7 | 1.24(0.35,4.40) | 0.7344 | 1.11(0.34,3.66) | 0.4336 |  |
| ≥75 | 955 | 15 | 973 | 16 | 1.31(0.62,2.76) | 0.4808 | 1.35(0.64,2.84) | 0.864 |  |
| Comorbidity |  |  |  |  |  |  |  |  | 0.4225 |
| <=3 | 668 | 8 | 671 | 5 | 1.05(0.33,3.37) | 0.9391 | 0.9(0.28,2.83) | 0.2684 |  |
| >3 | 738 | 13 | 735 | 18 | 1.57(0.73,3.38) | 0.245 | 1.54(0.72,3.3) | 0.8511 |  |
| Anticoagulant drug used within 3 month after the index-date | | | | |  |  |  |  | 0.9898 |
| Yes | 26 | 1 | 23 | 1 | 0.90(0.01,86.76) | 0.9637 | 0.44(0.00,52.43) | 0.7368 |  |
| No | 1380 | 20 | 1383 | 22 | 1.37(0.72,2.60) | 0.3342 | 1.33(0.7,2.53) | 0.3783 |  |
| Congestive Heart Failure | | |  |  |  |  |  |  | 0.8907 |
| Yes | 270 | 8 | 263 | 9 | 1.00(0.36,2.74) | 0.9947 | 1.25(0.46,3.35) | 0.6602 |  |
| No | 1136 | 13 | 1143 | 14 | 1.38(0.60,3.14) | 0.4488 | 1.35(0.59,3.06) | 0.4796 |  |

^§^Model was adjusted for all variables listed in Table 1.

^‡^ Model Adjusted for propensity score.

Abbreviations: aHRs, adjusted hazard ratios; CI, confidence interval.
